# Unveiling Antimicrobial and Insecticidal Activities of Biosynthesized Selenium Nanoparticles Using Prickly Pear Peel Waste

**DOI:** 10.3390/jfb13030112

**Published:** 2022-08-02

**Authors:** Amr H. Hashem, Tharwat A. Selim, Mohammed H. Alruhaili, Samy Selim, Dalal Hussien M. Alkhalifah, Soad K. Al Jaouni, Salem S. Salem

**Affiliations:** 1 Botany and Microbiology Department, Faculty of Science, Al-Azhar University, Cairo 11884, Egypt; 2 Zoology and Entomology Department, Faculty of Science, Al-Azhar University, Cairo 11884, Egypt; 3 Medical Microbiology and Parasitology Department, Faculty of Medicine, King AbdulAziz University, Jeddah 21589, Saudi Arabia; malruhaili@kau.edu.sa; 4 Department of Clinical Laboratory Sciences, College of Applied Medical Sciences, Jouf University, Sakaka 72341, Saudi Arabia; sabdulsalam@ju.edu.sa; 5 Department of Biology, College of Science, Princess Nourah bint Abdulrahman University, Riyadh 11671, Saudi Arabia; dhalkalifah@pnu.edu.sa; 6 Department of Hematology/Oncology, Yousef Abdulatif Jameel Scientific Chair of Prophetic Medicine Application, Faculty of Medicine, King Abdulaziz University, Jeddah 21589, Saudi Arabia; saljaouni@kau.edu.sa

**Keywords:** antibacterial activity, antifungal activity, insecticidal activity, selenium nanoparticles, prickly pear peel

## Abstract

In the current study, prickly pear peel waste (PPPW) extract was used for the biosynthesis of selenium nanoparticles through a green and eco-friendly method for the first time. The biosynthesized SeNPs were characterized using UV-Vis, XRD, FTIR, TEM, SEM, EDX, and mapping. Characterization results revealed that biosynthesized SeNPs were spherical, polydisperse, highly crystalline, and had sizes in the range of 10–87.4 nm. Antibacterial, antifungal, and insecticidal activities of biosynthesized SeNPs were evaluated. Results revealed that SeNPs exhibited promising antibacterial against Gram negative (*E. coli* and *P. aeruginosa*) and Gram positive (*B. subtilis* and *S. aureus*) bacteria where MICs were 125, 125, 62.5, and 15.62 µg/mL, respectively. Moreover, SeNPs showed potential antifungal activity toward *Candida albicans* and *Cryptococcus neoformans* where MICs were 3.9 and 7.81 µg/mL, respectively. Furthermore, tested crud extract and SeNPs severely induced larvicidal activity for tested mosquitoes with LC_50_ and LC_90_ of 219.841, 950.087 mg/L and 75.411, 208.289 mg/L, respectively. The fecundity and hatchability of *C. pipiens* mosquito were significantly decreased as applied concentrations increased either for the crude or the fabricated SeNPs extracts. In conclusion, the biosynthesized SeNPs using prickly pear peel waste have antibacterial, antifungal, and insecticidal activities, which can be used in biomedical and environmental applications.

## 1. Introduction

Multi-drug resistant bacteria have emerged due to the overuse or misuse of antibiotics. Additionally, the modern treatment of antibiotics in clinically prescribed dosages is not able to manage these pathogens; therefore, preventive strategies are necessary [1]. Likewise, fungi have become more resistant to common antifungal agents; thus, pathogenic fungi invade more than 1.2 billion individuals throughout the world, with at least 1.7 million deaths/year [2,3,4]. The mortality of fungal pathogens has become equal to drug-resistant *Mycobacterium tuberculosis*, exceeding malaria [5]. The recent annual incidence of invasive candidiasis was 750,000 cases, respectively [6].

Furthermore, mosquitoes have caused many problems for humans; the most common mosquito vector across tropical and subtropical regions is *Culex* sp. It is the main vector of lymphatic filariasis, which has recently been reported to affect approximately 51 million individuals globally [7]. *Culex pipiens* (L.) has worldwide distribution, resulting in many pathogens [8,9]. Mosquito control is essential to prevent the spread of mosquito-borne diseases and to improve the quality of breeding and public health. The basic strategy for mosquito control involves using chemical insecticides, such as organophosphate and organochlorine compounds. However, this has not been very successful due to ecological and economic factors [10]. Recently, plant sources have become a promising alternative to synthetic chemical agents for vector control [11].

Today’s antimicrobial materials utilized in clinical settings suffer from serious flaws, such as poor antimicrobial activities, danger in microbial resistance, difficulties monitoring and improving antibacterial functionalities, and trouble operating in a dynamic environment. Additionally, there is a significant chance that microbes will develop resistance to traditional antibiotics. As a result, several antimicrobial protection strategies are needed.

Nanotechnology has the potential to revolutionize a wide array of applications in the fields of catalysis, sensors, optoelectronics, drug delivery, antimicrobial agents, vector control, and parasitology [12,13,14,15,16,17,18,19,20,21,22,23,24]. Recent years have seen the rapid synthesis of nanocrystals (Ag, Zn, Se, Au, and Cu) using biological (plants, fungi, algae, and bacteria) techniques [25,26,27,28,29,30,31,32]. These NPs are prepared in pure, non-toxic, and environmentally friendly manners by utilizing high-energy renewable materials to support the safety and reliability of the NP development processes [33,34,35,36,37,38,39]. Plant-fabricated NP development can proceed more quickly since there is no need to maintain the exact conditions in media and culture that are necessary for other biological entities [40,41,42]. In order to act as reductive and stabilizing agents, co-factors, such as enzymes, terpenoids, flavonoids, and proteins are included in plant extracts [43]. Green pathway NPs often have strong catalytic abilities because of their high surface areas and capacities to boost reactivity by creating reactive-oxygen species, which cause increased toxicity in bacteria cells and carcinomas. One of the essential trace elements that mammals and higher animals need for appropriate cellular function is selenium [44]. Due to its low toxicity and great stability, selenium Nano (SeNPs) are now embraced by a large number of researchers and are suggested for use in a variety of scientific fields [45,46,47]. In a dose-dependent approach, SeNPs have demonstrated antibacterial action against both conventional and antibiotic-resistant variants of Gram negative and Gram positive bacteria [48]. Due to interactions between selenium nanoparticles and various protein molecular structures, they have high adsorption and microbial properties. Wadhwani et al. claimed that biologically-generated SeNPs are simpler, more environmentally friendly, and economically feasible than alternative methods (chemically and physically) [49]. Publications on biological methods used to generate SeNPs (using plant cell parts, such dried leaves, seeds, and flowers) are available [48,50].

The main goal of the current study was to quickly create SeNPs from prickly pear peel waste (PPPW) extract and assess their potential for use in a variety of applications, including (i) antibacterial activity, (ii) antifungal activity, (iii) toxicity bioassay against *C. pipiens* mosquito larvae, and (iv) its impact on the mosquito’s fecundity and ability to hatch eggs.

## 2. Materials and Methods

### 2.1. Materials

The waste of prickly pear peel was collected from a marketplaces in Giza, Egypt. Na_2_SeO_3_, nutrient agar, Muller Hinton agar, and potato dextrose agar media were purchased from Sigma Aldrich, Darmstadt, Germany.

### 2.2. Methods

#### 2.2.1. Preparation of Prickly Pear Peel Waste (PPPW) Extract

The waste of prickly pear peel was gathered from marketplaces in Giza, Egypt. The obtained samples were brought to the lab; processing started right away. For PPPW, no disease signs were chosen, and they underwent two rounds of distilled water washing. The peel was then broken up into small pieces (about 1 cm), placed in a 2 L Erlenmeyer flask with 1000 mL of sterile distilled water, and blended for 3 min at 1000 rpm in a mixing bowl (mixer). After filtering via Whatman No. one filter paper, the mixture was collected in a purified bottle and preserved at 4 °C until it was used.

#### 2.2.2. Biosynthesis of SeNPs

The purified PPPW extract was used to generate SeNPs. In a clean Erlenmeyer flask, 10 mL of PPPW extract and 90 mL of 2 mM Na_2_SeO_3_ solution were combined to make the appropriate reaction mixture. On the other hand, as a control, the identical experimental setup containing 10 mL PPPW extract with 90 mL of distilled water was used. For three hours in the dark, both flasks were incubated in the orbital shaker to produce a homogenous solution. Afterward, dist. water and centrifuge were used to separate and purify the produced SeNPs. For further analysis and bioactivity evaluation, the dried SeNPs were stored at room temperature.

#### 2.2.3. Characterization of SeNPs

The SeNPs were characterized using a range of instrumental analysis tools. Visual inspection of SeNP formation throughout the incubation period was conducted using variations in the solution color.

##### UV–Vis Spectroscopy

The UV-visible spectroscopy (JENWAY-6305 Spector, JENWAY, Staffordshire, UK) was used to examine the optical properties of the SeNPs between 200 and 800 nm.

##### Fourier-Transform Infrared (FT-IR) Spectroscopy

The FT-IR spectra of the prepared sample were measured on a Spectrum Two-IR Spectrometer (PerkinElmer Inc., Shelton, DC, USA). The records were conducted in the range of 400–4000 cm^−1^ with a resolution of 4 cm^−1^ and 32 scans.

##### X-ray Diffraction (XRD) Spectroscopy

The XRD patterns of the generated SeNPs were examined using a Diano-X-ray diffractometer (Philips) equipped with a Cu-K source of radiation (λ = 0.15418 nm) active at 45 kV, a generator (PW, 1930), and a goniometer (PW, 1820).

##### Transmission and Scanning Electron Microscopy

The TEM technique was used to determine the generated SeNPs sizes and morphology. A 200 kV voltage was used, using the ultra-high resolution TEM (JEOL-2010, Akishima, Tokyo). To make TEM grids, a drop of the particle solution was applied to a copper grid with carbon coating and allowed to dry while illuminated. Moreover, SEM analysis (SEM, ZEISS, EVO-MA10, Jena, Germany) was used to elucidate the surface morphology, boundary size, and the distribution of the synthesized SeNPs. To study the elemental composition, purity, simplicity, and the distribution of element shape-prepared SeNPs, the EDX, BRUKER, Nano GmbH, D-12489, 410-M, Berlin, Germany, was employed. 

#### 2.2.4. Antimicrobial Activity

Antimicrobial activity of biosynthesized SeNPs was assessed using the agar well diffusion technique against bacterial strains (*Staphylococcus aureus* ATCC 25923, *Bacillus subtilis* ATCC605, *Escherichia coli* ATCC 25922, *Pseudomonas aeruginosa* ATCC 27853), and unicellular fungal strains (*Candida albicans ATCC90028*, *Cryptococcus neoformans* ATCC 14116). The bacterial strains were cultured on nutrient broth media for 24 h at 37 °C. Bacterial suspensions of 1.5 × 10^6^ CFU/mL were separately prepared, seeded into Muller Hinton agar media, and poured aseptically into sterilized petri plates. Moreover, 100 µL of SeNPs (2000 µg/mL), PPPE, and standard antibiotic (Ampicillin/sulbactam) were added in agar well, and then plates were put in the refrigerator for 2 h followed by incubation at 37 °C for 24 h. Fungal strains were initially grown on PDA plates and incubated at 30 °C for 3–5 days. As well, unicellular fungi suspensions were seeded separately; many wells (7 mm) were made, and then 100 µL of SeNPs (2000 µg/mL), PPPE, and reference antifungal (amphotericin B) were added. All PDA plates were incubated at 30 °C for 48 h and then the inhibition zone diameter was measured. To determine the minimum inhibitory concentration, SeNPs were prepared in different concentrations ranging from 2000 to 1.95 µg/mL, and then assessed separately to detect MIC against selected bacterial and fungal strains [51,52].

#### 2.2.5. Insecticidal Activity

##### Mosquito Colony

A mosquito colony of *Culex pipiens* was purchased from the Medical Entomology Institute (Cairo, Egypt). Later, it was reared for many generations at the Insectary of Medical Entomology, the Department of Zoology and Entomology, Faculty of Science, Al-Azhar University, under optimum conditions

##### Larvicidal Activity

The larvicidal property of the PPPW extract and SeNPs was estimated against the *C. pipiens* mosquito, according to WHO 2005. Around 25 larvae from the third instar of the tested mosquito were picked up and placed in plastic cups (500 mL capacity) containing 250 mL tap water (249 mL of water and 1 mL of the tested concentration). The PPPW extract was tested to compare results with others of nanoparticles with concentrations of 50, 100, 200, 400, and 800 mg/L. Synthesized SeNPs were added at serial concentrations of 25, 50, 100, 200, and 400 mg/L. Each concentration was tested in triplicates. The control was tested alongside for both samples at the same conditions. Mortality percentages were recorded 24 h post-test.

##### Fecundity and Hatchability

For each concentration, equal numbers of males and females succeeded to survive and emerge from each treatment alongside the control were transferred to 30 × 30 × 30 cm wooden cadges. Deposited eggs were collected from plastic cups three days post-feeding daily. Fecundity was calculated by counting the total number of eggs laid divided by the number of females that mated and survived until the end of the experiment.

The hatchability percentage was estimated according to the Hassan et al. [53] equation,

Hatchability% = A/B × 100

where A = Total No. of hatched eggs; B = Total No. of laid eggs.

#### 2.2.6. Statistical Analysis

Descriptive statistics, including mean and standard error (SE), were calculated for each treatment. The mean larval mortality data were subjected to probit analysis to calculate the Chi-square value and LC50 and LC90 at 95% confidence limits. The one-way analysis of variance, lower and upper confidence limits, and Chi-square values were conducted using SPSS (ver. 25). The LSD post hoc test was used for pairwise comparisons. Na_2_SeO_3_ treatments revealed zero larval mortality. Data are presented as mean ± SE. The *p* value was considered significant at <0.05.

## 3. Results and Discussion

### 3.1. Biosynthesis and Characterization of SeNPs

**Figure 1:**
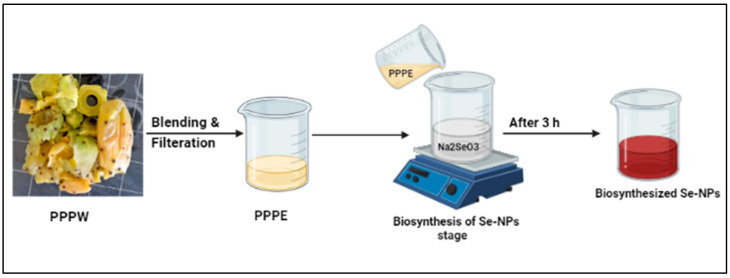
Green biosynthesis of SeNPs using PPPE.

Due to their unique uses in nanotechnology, materials obtained from plants are typically regarded as sustainable, environmentally friendly, and having economic worth. The creation of nanoparticles has traditionally involved the use of chemical and physical processes, but recently, biological processes have attracted a lot of attention [54]. The interaction of the PPPW extract with selenite caused the solution to become reddish, indicating the synthesis of SeNPs. This interaction demonstrated the ability of the PPPW extract’s constituents to reduce the selenium ions and transform them into SeNPs. In order to produce environmentally acceptable SeNPs, prickly pear peel extract was used as a stabilizing and reductive agent. As indicated by a gradual change in the solution color from pale-yellow to deep red, suggesting SeNPs biosynthesis, Na_2_SeO_3_ was bio-reduced to SeNPs by employing PPPW (Figure 1). Citrus fruit extract was employed by Alvi et al. [55] to create SeNPs. Comparable color changes from yellow to reddish served as evidence that SeNPs had formed.

#### 3.1.1. UV–Vis Spectroscopy

**Figure 2:**
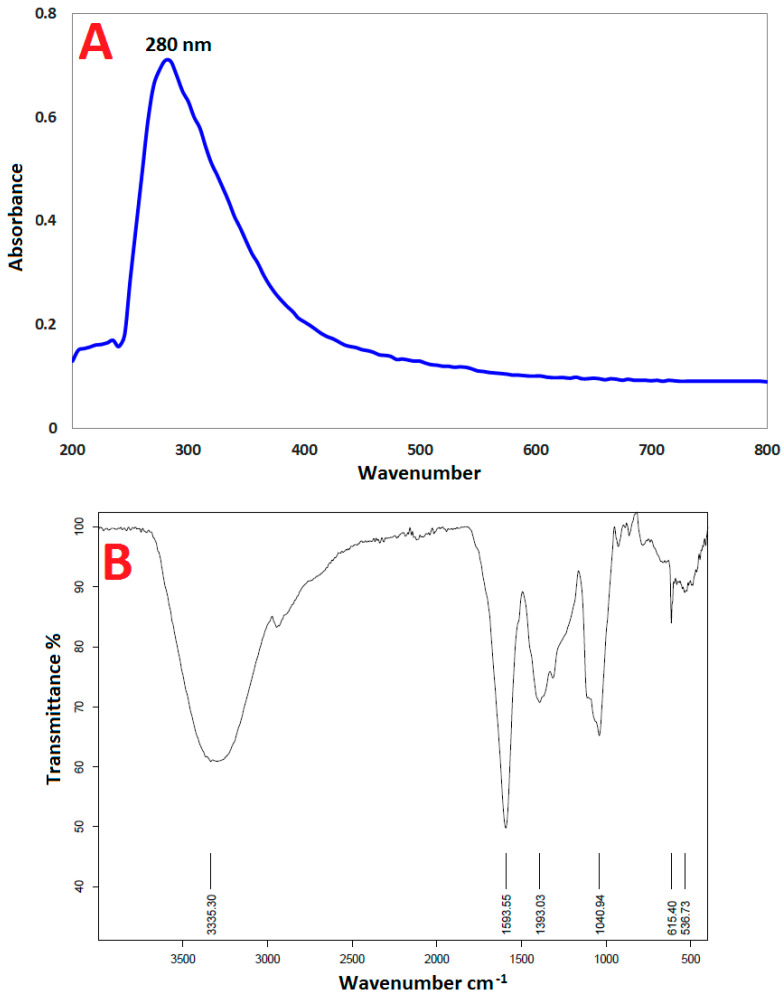
(**A**) UV–Vis spectrum and (**B**) FTIR analysis of SeNPs prepared from PPPW extract.

The UV analysis of SeNPs produced by PPPW is shown in Figure 2A, and it exhibits a prominent peak at 280 nm. Due to the SeNP formation surface plasmonic resonating (SPR ) peak that may be shown as a broad emission spectrum in the range of wavelength of 270–400 nm, the SeNP formation could be clearly validated using the UV–Vis analysis [52,56,57]. In their UV–Vis spectra, SeNPs exhibit a noticeable peak at around 280 nm, which is attributed to spherical SeNPs [58,59]. The produced NPs are easy to make and stable, and the green syntheses of SeNPs are simple and safe for the environment. Selenium nanoparticles were synthesized by extracts of different plant parts [41,60]. The rarity of using prickly pear peel extract in the preparation of SeNPs highlights the originality of our study on SeNP biosynthesis from PPPW. 

#### 3.1.2. Fourier-Transform Infrared (FT-IR) Spectroscopy

Research using FT-IR spectroscopy was also conducted to confirm the potential role of PPPW extract in SeNP production. FT-IR can identify the functional groups that are present on the SeNPs surface by identifying the excitations of chemical bonds. Finding conformational changes in the coordination identity of organic biomolecules on SeNPs surfaces is made easy by the chemical information obtained. Wave numbers at 3335.30 cm^−1^, 1593.55 cm^−1^, 1393.03 cm^−1^, 1040.94 cm^−1^, 615.40 cm^−1^, and 536.73 cm^−1^ represent the capping agent from PPPW extract interacting with SeNPs (Figure 2B). The line in the spectra at 3335.30 cm^−1^ corresponds to –O-H stretch vibrates, indicating that alcohol and phenol groups are present in the PPPW extract [55]. The peak at 1593.55 cm^−1^, which correlates with -N-C- and -C-C- stretching, indicates the presence of proteins. The -N-H stretch resonance seen in the am-ide bonds of the proteins was linked to the spectra at 1393.03 cm^−1^ in the spectrum. Proteins N-H and C-N (amines) have stretch vibrations that were detected in their spectra at 1393.03 cm^−1^ and 1040.94 cm^−1^, respectively. Peaks at 615.40 cm^−1^ and 536.73 cm^−1^ in the FTIR portions of the SeNPs spectra were attributed to the binding of SeNPs with PPPW extract-prepared biomaterials. According to FTIR investigations, proteins and carbohydrates were shown to be the most prevalent substances on the SeNPs surface. The differences in the peaks indicate that organic components in the PPPW extract facilitated the synthesis of SeNPs during the reduction process effectively. These components may also assist prevent SeNPs from aggregating and preserve their long-term stability [13,55].

#### 3.1.3. X-ray Diffraction (XRD) Spectroscopy

**Figure 3:**
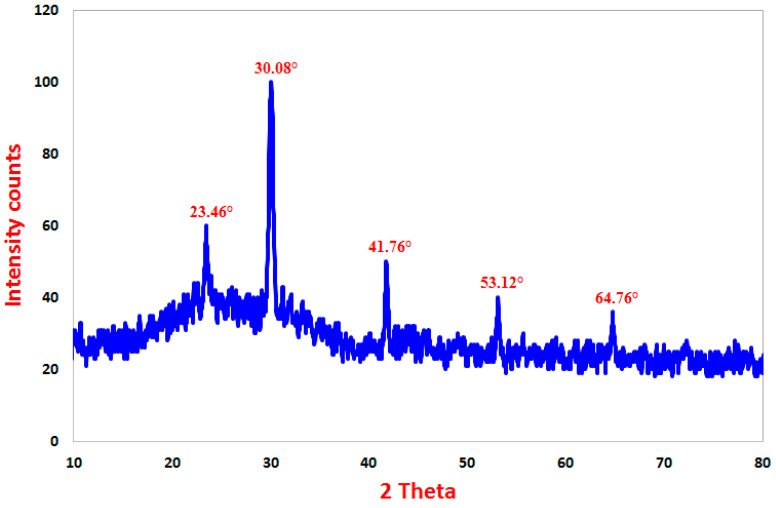
XRD pattern of SeNPs prepared by PPPW extract.

Crystal structure and phase of the prepared SeNPs were analyzed using XRD analysis. The XRD pattern of the synthesized SeNPs is presented in Figure 3. It is clearly shown in the pattern that there are no characteristic peaks for the starting precursors. SeNPs XRD diffraction peaks are shown in Figure 3, along with the diffraction characteristics regarding 2θ at 23.46°, 30.08°, 41.76°, 53.12°, and 64.76°, which represent the Bragg’s reflections at (100), (101), (111), (201), (210), respectively. The Joint Committee on Powder-Diffraction Standards (JCPDS) of SeNPs using a reference card-JCPDS no. 06-0362 showed that all of the peaks were comparable [61]. According to our explanation of the findings, earlier research showed that plant extract mediators may be used to successfully fabricate crystallite, cubic phase form SeNPs at the same XRD diffraction planes [62,63]. The XRD results indicate that the produced SeNPs were highly-crystalline for better application.

#### 3.1.4. Transmission and Scanning Electron Microscopy

**Figure 4:**
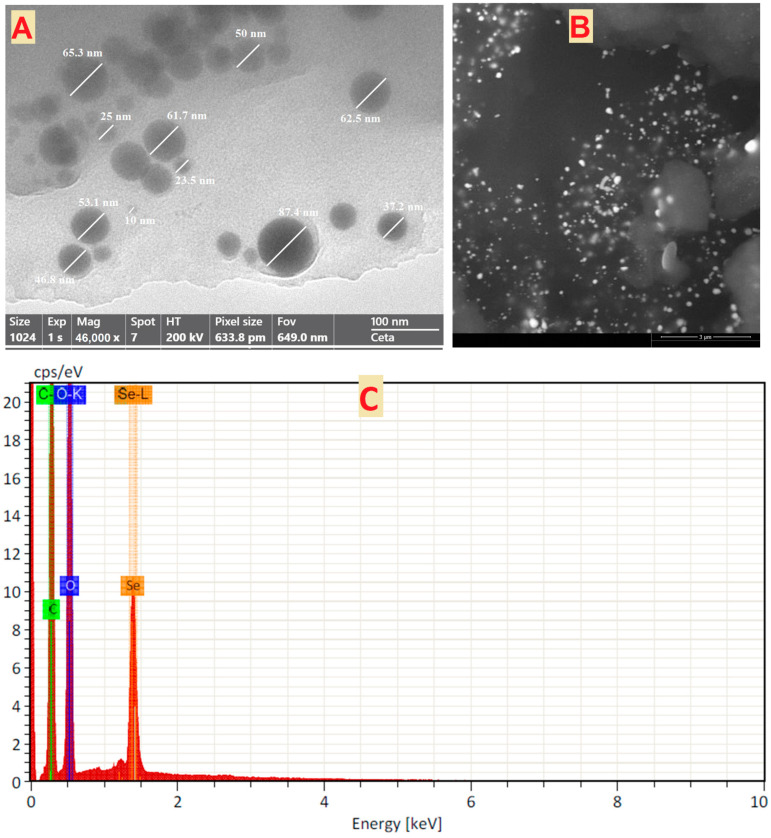

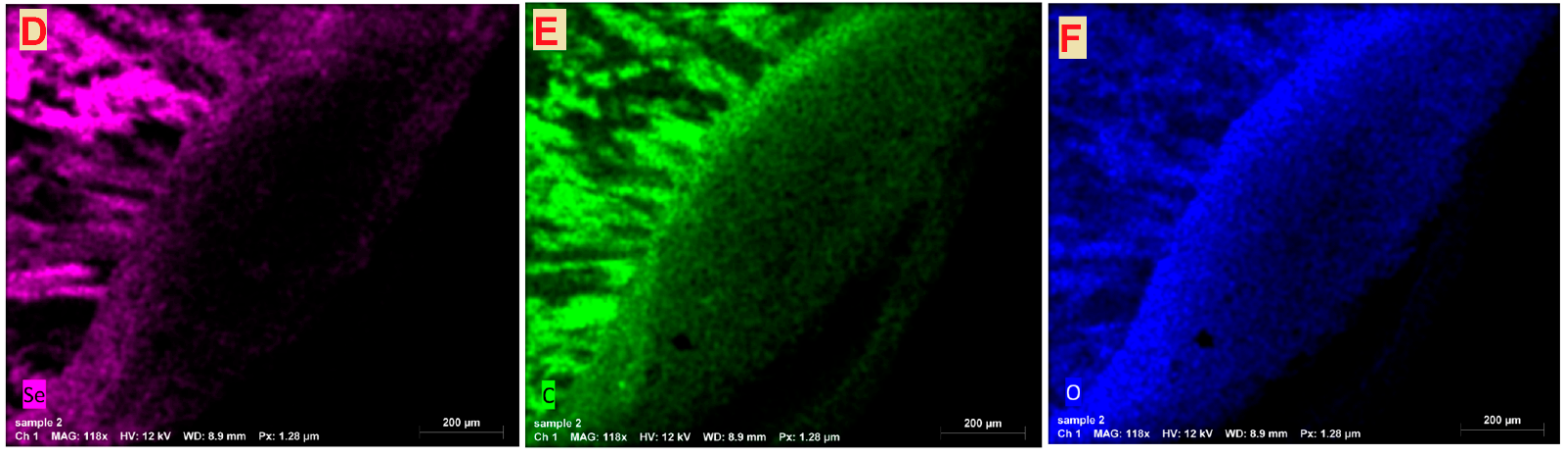
(**A**)TEM image, (**B**) SEM image, (**C**) elemental analysis, and (**D**–**F**) SEM/EDX mapping of SeNPs prepared by PPPW extract.

According to the TEM image, the SeNPs were spherical and ranged in size from 10–87.4 nm (Figure 4A). Additionally, TEM micrographs showed that the SeNPs were dispersed uniformly. The morphologies of the surface and particle sizes of SeNPs were assessed using the SEM, as seen in Figure 4B. SeNPs had almost spherical shapes. The size of the Se-Nano produced from extract of the plant in a colloidal at room temperature ranges from 50 to 150 nm [64]. Shakibaie et al. also generated spherical SeNPs with the highest frequencies of 120–140 nm inside the range of 80–220 nm [65]. The SeNP size that was produced in this study is preferred above the SeNP sizes that were shown in the prior results, which ranged from 100 to 500 nm [66], The SeNP powder’s elemental composition was ascertained via the EDX analysis [67]. The EDX spectra of the SeNPs showed the presence of multiple distinct elements linked to the selenium, oxygen, and carbon components (Figure 4C). Carbon and oxygen may be coated on the SeNPs [60]. In the mapping, the “carbon” and “oxygen” relate to the PPPW extract, while the “selenium” refer to the synthesis of SeNPs (Figure 4D–F). These outcomes are consistent with previous studies [68,69]. 

### 3.2. Antimicrobial Activity

**Figure 5:**
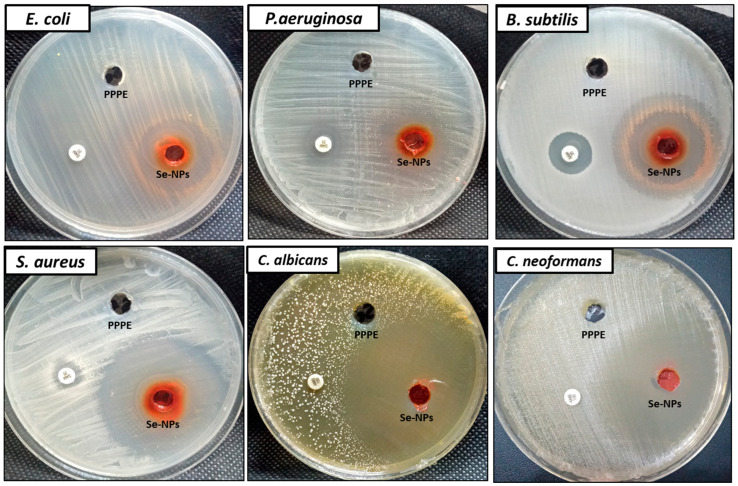
Antimicrobial activity of biosynthesized SeNPs using the agar well diffusion method.

Antimicrobial activity of biosynthesized SeNPs was evaluated against Gram negative, Gram positive bacteria, and unicellular fungi, as shown in Figure 5 and Table 1. Results revealed that the biosynthesized SeNPs showed antibacterial activity as well as antifungal activity against the tested bacterial and fungal strains. Moreover, biosynthesized SeNPs showed antibacterial activity against Gram positive bacteria higher than Gram negative bacteria, where inhibition zones of SeNPs (2000 µg/mL) against *B.* *subtilis* and *S. aureus* were 30.7 ± 0.53 and 48.7 ± 1.06 mm, respectively, while 26.5 ± 070 and 24.4 ± 0.85 mm toward *E. coli* and *P. aeruginosa* respectively. Furthermore, different concentrations of SeNPs were evaluated for antibacterial activity against all tested bacterial strains. The obtained result revealed that concentrations 125–2000 µg/mL exhibited antibacterial activity against *E. coli* and *P. aeruginosa*, this indicates MIC was 125 µg/mL. In the case of *B. subtilis* and *S. aureus*, MIC was 62.5 and 15.62 µg/mL respectively.

Likewise, biosynthesized SeNPs exhibited promising antifungal activity against unicellular fungi (*C. albicans* and *C. neoformans*) as shown in Table 1 and Figure 5. Results showed that inhibition zones of SeNPs (2000 µg/mL) against *C. albicans* and *C. neoformans* were 59.5 ± 0.70 and 50. 2 ± 1.13 mm respectively. Moreover, MIC_s_ of SeNPs toward *C. albicans* and *C. neoformans* were 3.9 and 7.81 µg/mL, respectively. From these data, the biosynthesized SeNPs from PPPW have antifungal activity at very low concentrations. Previous studies confirmed that SeNPs synthesized by plant extracts have antibacterial and antifungal activities [70,71]. Hashem and Salem [70] reported that the biosynthesized SeNPs from *Urtica dioica* (stinging nettle) leaf extract have antibacterial and antifungal activities against human pathogenic bacterial and tested bacterial strains, where MICs against bacterial strains were in the range of 31.25–500 µg/mL, while MICs toward fungal strains were in the range of 7.81–31.25 µg/mL. Moreover, Lashin et al. [71] confirmed SeNPs that biosynthesized from *Ziziphus spina-christi* exhibited antimicrobial activity against Gram negative bacteria, Gram positive bacteria, and unicellular and multicellular fungi. Alvi et al. [55] prepared SeNPs from *Citrus limon* (lemons) and *Citrus paradise* (grapefruits) extracts and found it had antibacterial activity against *E. coli*, *Micrococcus luteus*, *B. subtilis*, and *Klebsiella pneumonia*. Salem et al. [72] reported that the biosynthesized SeNPs from pomegranate peel extract have promising antifungal activity. Huang et al. [73] suggested four mechanisms for SeNP-mediated antimicrobial properties, which include depolarization, disturbances of biological membrane, interruption of intracellular adenosine triphosphate (ATP) levels, and the reactive oxygen effect. 

### 3.3. Insecticidal Activity

Mosquito-borne diseases are one of the main public health issues in developing countries. Usage of synthetic insecticides as mosquitocidals has become complicated due to mosquito resistance, toxicity to humans, and non-targeted organisms, all of this has stimulated interest in searching for novel control methods [74]. Plant extracts have been regarded as alternative agent insecticides [75].

Increasing evidence suggests that green-fabricated mosquitocidal nanoparticles may be more effective than the plant extracts tested alone [59,76]. There are few studies on nanoparticle toxicity concerning filariasis vector *C. pipiens* [77]. Concerning the mechanism of action, we hypothesized that the toxicity of green-fabricated nanoparticles on mosquitoes could be owing to the nanoparticles’ small sizes, which allow them to penetrate cuticle insects.

The third instar larvae of tested *C. pipiens* mosquito was treated with five concentrations of both the crude PPPW extract of *Opuntia ficus-indica* (50, 100, 200, 400, and 800 mg/L) and synthesized *SeNPs* (25, 50, 100, 200, and 400 ppm). Based on the obtained results in (Table 2), the recorded LC50 and LC90 values for the crude PPPW extract were (219.841 mg/L) and (950.087 mg/L) while SeNPs were (75.411 mg/L) and (208.289 mg/L), respectively. The highest larvicidal activity percentage (100) was achieved when synthesized *SeNPs* were 400 mg/L compared with the control group, with no larval mortality recorded in the group treated with SeNPs alone (without plant extract). Our results revealed that larvicidal effects of both *Opuntia ficus-indica* and green-synthesized *SeNPs* conducted on *C. pipiens* mosquitoes had toxicity with LC50 and LC90 values (219.841, 950.087 mg/L) and (75.411, 208.289 mg/L), respectively. The highest larvicidal activity percentage (100) was achieved when synthesized *SeNPs* at concentrations of 400 mg/L compared with the control group.

Selenium-nanoparticle synthesized utilization plant extracts have great biological activity and adsorption capacity due to the overlap between selenium at the nano-scale and the NH, C=O, COO, and C-N functional protein groups [78]. A recent study proved that the SeNPs synthesized employ *Penicillium corylophilum*—a good agent in mosquitos controlling, cell lines, and Gram positive and Gram negative bacteria [79]. A new study reported that selenium nanoparticles synthesized from Clausena dentate were a good agent against different mosquito species with different concentrations [80], Whereas, in Krishnan et al.’s [81] study, SeNPs synthesized from *D. indica* were more effective by controlling early instar larvae of *C. quinquefasciatus* and *A. aegypti* at different concentrations. The toxicity of biosynthesized selenium nanoparticles proved to be just as good as that of gold and silver nanoparticles [82] in controlling both *C. quinquefasciatus* and *A. aegypti*. The toxicity of selenium nanoparticles manufactured using the leaf extract of *D. indica* to the larvae and pupae of both types of mosquitoes may be due to the intracellular toxic effects of intradermal nanoparticles and other peripheral cells. Furthermore, the toxicity of SeNPs may be due to denaturation of organelles and enzymes that reduce membrane permeability, further affecting ATP synthesis and ultimately inhibiting cellular function, leading to death [83]. However, many other relevant reports of natural extracts, such as plant nano-products as insecticides, have been reported. *Lagenaria siceraria* and ZnO-NPs mediated enhanced larvicide activity of *Anopheles stephensi* with an LC50 score of 56.46 ppm [84]. Hasaballah et al. [85] reported that So-ZnO-NPs had the larvicidal activity against the tested mosquitoes, this is attributed to the small sizes of the manufactured nanoparticles that allow easy penetration into insect skin and cells where it interferes with molting and other physiological processes [86]. Vinotha et al. [87] reported that cardamom-coated ZnO-NPs was a high potential agent against *Culex tritaeniorhynchus* with an LC50 of about 15.09 μg/mL.

Fecundity and hatchability of *C. pipiens* mosquito were significantly decreased as applied concentrations increased either for the crude or the fabricated *SeNP* extracts. The minimum recorded fecundity and hatchability were achieved with the concentration of 400 mg/L of *SeNP* extracts, where both decreased to more than 90% from those of the control group (Table 3). The effects were much more pronounced in the *SeNP* extract than in the crude extract. The current results also revealed that fecundity and hatchability of the *C. pipiens* mosquito significantly decreased as applied concentrations increased either for the crude or the fabricated *SeNPs* extracts. The minimum recorded fecundity and hatchability were achieved at 400 mg/L of *SeNP* extract where both decreased to more than 90% from those of the control group; the effects were much more pronounced in the *SeNP extract* than the crude extract. Fecundity was inversely proportional to the applied concentrations of the crude extract of *S. officinalis* and *So*-ZnO-NPs [84].

Hasaballah et al. [11] revealed that So-ZnO-NPs at a concentration of 80 ppm exhibited decreasing fertility of *C. pipiens* females by more than 50%, and this effect was more pronounced in *An. pharoensis* females at concentrations greater than 40 ppm compared to the untreated group. In the same vein, Roni et al. [88] showed that concentrations ranging from 100 to 500 ppm of Hypnea musciformis-synthesized AgNP severely reduced the fecundity of the *Ae. Aegypti* female. Furthermore, Madhiyazhagan et al. [89] found that *S. Muticum*-synthesized AgNP decreased the oviposition rates to more than 70% in *C. quinquefasciatus*, *Ae. Aegypti*, and *An. Stephensi* when treated with a concentration of 10 ppm. In the same context, egg hatchability of *A. aegypti*, *A. stephensi*, and *C. quinquefasciatus* was reduced by 100% after treatment with 30 ppm of AgNP, while control eggs showed 100% hatchability [89].

## 4. Conclusions

In this study, SeNPs were biosynthesized from PPPW extract for the first time through a green and ecofriendly method. The biosynthesized SeNPs were characterized using various modern techniques. Characterization results revealed that SeNPs were highly crystalline, and spherical in shape. Moreover, the biosynthesized SeNPs exhibited promising antibacterial and antifungal activity against Gram negative and Gram positive bacteria and unicellular fungi, which cause many problems to humans. Furthermore, SeNPs had larvicidal activity as well as decreased the fecundity and hatchability of the *C. pipiens* mosquito. Therefore, the biosynthesized SeNPs in this study can be used in medical and environmental applications where they have antimicrobial as well as insecticidal activities.

## Figures and Tables

**Table 1:** Antimicrobial activity of SeNPs at different concentrations.

Test Material		*E. coli*	*P. aeruginosa*	*B. subtilis*	*S. aureus*	*C. albicans*	*C. neoformans*
SeNPs (µg/mL)	2000	26.5 ± 0.70 ^a^	24.4 ± 0.85 ^a^	30.7 ± 0.53 ^a^	48.7 ± 1.06 ^a^	59.5 ± 0.70 ^a^	50.2 ± 1.13 ^a^
	1000	21.7 ± 1.06 ^b^	21.6 ± 0.56 ^b^	27.5 ± 0.35 ^b^	41.5 ± 0.70 ^b^	54.7 ± 0.99 ^b^	46.4 ± 0.84 ^b^
	500	15.4 ± 0.56 ^c^	17.6 ± 0.85 ^c^	23.6 ± 0.46 ^c^	34.8 ± 1.20 ^c^	51.0 ± 1.34 ^c^	40.85 ± 1.20 ^c^
	250	11.7 ± 0.35 ^d^	11.3 ± 0.49 ^d^	19.2 ± 0.17 ^d^	29.6 ± 0.56 ^d^	45 ± 1.41 ^d^	36.8 ± 1.13 ^d^
	125	8.2 ± 0.35 ^e^	8.7 ± 0.42 ^e^	13.5 ± 0.32 ^e^	24.6 ± 0.84 ^e^	37.3 ± 0.91 ^e^	30.4 ± 0.64 ^e^
	62.5	0 ± 00 ^f^	0 ± 00 ^f^	8.7 ± 0.21 ^f^	20.7 ± 0.84 ^f^	34.25 ± 0.35 ^f^	26.4 ± 0.84 ^f^
	31.25	0 ± 00 ^f^	0 ± 00 ^f^	0 ± 00 ^g^	16.5 ± 0.70 ^g^	27.5 ± 0.70 ^g^	20.1 ± 1.20 ^g^
	15.62	0 ± 00 ^f^	0 ± 00 ^f^	0 ± 00 ^g^	10.6 ± 0.92 ^h^	24 ± 1.41 ^h^	14 ± 1.41 ^h^
	7.81	0 ± 00 ^f^	0 ± 00 ^f^	0 ± 00 ^g^	0 ± 00 ^i^	16.5 ± 0.70 ^i^	10.7 ± 0.35 ^i^
	3.9	0 ± 00 ^f^	0 ± 00 ^f^	0 ± 00 ^g^	0 ± 00 ^i^	12.3 ± 0.91 ^j^	0 ± 00 ^j^
	1.95	0 ± 00 ^f^	0 ± 00 ^f^	0 ± 00 ^g^	0 ± 00 ^i^	0 ± 00 ^k^	0 ± 00 ^j^
PPPW *		0±00	0 ± 00	0 ± 00	0 ± 00	0 ± 00	0 ± 00
SAM/AMB **		0±00	14.5 ± 0.5	15.6 ± 0.4	10.1 ± 0.9	0 ± 00	0 ± 00

* PPPE means prickly pear peel and ** SAM/AMB means (Ampicillin/sulbactam)/Amphotericin B. Superscript letters from a to k revealed the power of significance.

**Table 2:** Larvicidal activity of the PPPW crude and synthesized Opuntia ficus-indica Selenium nanoparticles (SeNPs) extracts for the mosquito vector, *C. pipiens*.

Treatments	Concentrations (mg/L)	*n*	Larval Mortality % ± SD	LC_50_ (LCL–UCL) (mg/L)	LC_90_ (LCL–UCL) (mg/L)	Statistic Summary
Crude extract of PPPW	Control	75	0.0 ± 0.0 ^a^	219.841 (186.330–260.524)	950.087 (715.547–1407.422)	*d*. *f*. = 5, F = 800.96, *p* < 0.001, χ^2^ = 2.396
	50	75	10.67 ± 1.33 ^b^			
	100	75	26.67 ± 1.33 ^c^			
	200	75	41.33 ± 1.33 ^d^			
	400	75	69.33 ± 1.33 ^e^			
	800	75	89.33 ±1.33 ^f^			
SeNPs	Control	75	0.0 ± 0.0 ^a^	75.411 (66.163–85.797)	208.289 (173.006–265.562)	*d*. *f*. = 5, F = 254.757, *p* < 0.001, χ^2^ = 13.331
	25	75	13.3 ± 1.33 ^b^			
	50	75	28.0 ± 4.0 ^c^			
	100	75	52.0 ± 4.61^d^			
	200	75	93.3 ± 2.58 ^e^			
	400	75	100.0 ± 0.0 ^e^			
Se ions	Nil	Nil	Nil	Nil	Nil	Nil

Larval mortalities are presented as Mean ± SE of three replicates. Means with different letters are significantly different at (*p* < 0.05). (LC50) concentration that kills 50% of population, (LC90) concentration that kills 90% of population, (LCL) lower confidence limit, (UCL) upper confidence limit, (*d*. *f*.) degree of freedom, (χ^2^) Chi-square, *n* = sample size.

**Table 3:** Fecundity and egg-hatchability of the mosquito, *C. pipiens* treated with different concentrations of the PPPW crude extract, and SeNPs of *Opuntia ficus-indica*.

Treatment	Concentration (mg/L)	Fecundity Mean ± SE	Hatchability Mean ± SE	Hatchability (%)
Crude extract of PPPW	Control	142.0 ± 6.11 ^a^	138.3 ± 5.24 ^a^	97.46
	50	133.3 ± 4.41 ^a^	124.0 ± 4.16 ^b^	93.02
	100	110.0 ± 1.54 ^b^	93.3 ± 2.40 ^c^	84.9
	200	93.3 ± 1.67 ^c^	76.0 ± 1.0 ^d^	81.52
	400	59.3 ± 2.96 ^d^	44.67 ± 2.60 ^e^	75.23
	800	48.3 ± 1.67 ^e^	29.3 ± 2.40 ^f^	60.74
Statistic Summary		*p* < 0.001, *d*. *f*. = 5	*p* < 0.001, *d*. *f*. = 5 F = 172.918	
SeNPs	Control	143.67 ± 1.85 ^a^	131.67 ± 6.01 ^a^	91.577
	25	126.67 ± 1.67 ^b^	106.67 ± 1.67 ^b^	84.205
	50	98.0 ± 2.0 ^c^	72.33 ± 1.45 ^c^	73.929
	100	63.3 ± 1.67 ^d^	26.67 ± 1.76 ^d^	42.137
	200	30.67 ± 2.96 ^e^	10.0 ± 0.58 ^e^	32.893
	400	13.67 ± 1.85 ^f^	2.67 ± 0.88 ^f^	18.472
Statistic summary		*p* < 0.001, *d*. *f*. =5	*p* < 0.001, *d*. *f*. =5 F = 380.721	

Larval mortalities are presented as Mean ± SE of three replicates. Means with different letters are significantly different at (*p* < 0.05). (LC50) concentration that kills 50% of population, (LC90) concentration that kills 90% of population, (LCL) lower confidence limit, (UCL) upper confidence limit, (*d*. *f*.) degree of freedom, (χ^2^) Chi-square, *n* = sample size.

## Data Availability

Not applicable.
